# Supplementary material for: Sub-wavelength patterned pulse laser lithography for efficient fabrication of large-area metasurfaces
Source: Nat Commun. 2022 Oct 3;13:5823. doi: 10.1038/s41467-022-33644-8 (PMC9530239; doi:10.1038/s41467-022-33644-8)
Supplement: Supplementary file 1 — Supplementary information [file 41467_2022_33644_MOESM1_ESM.pdf]

Supplementary information for "Sub-wavelength patterned pulse laser lithography for efficient fabrication of large-area metasurfaces"

Lingyu Huang[1], Kang Xu[1], Dandan Yuan[1], Jin Hu[1], Xinwei Wang[2], Shaolin Xu[1,*]

[1]Department of Mechanical and Energy Engineering, Southern University of Science and Technology, 1088 Xueyuan Avenue, Shenzhen, 518055, China

[2]Department of Mechanical Engineering, Iowa State University, Ames, IA, 50011, United States

These authors contributed equally: Lingyu Huang and Kang Xu.

[*]Corresponding author. Email: xusl@sustech.edu.cn

1. Comparison of laser ablation between CGH calculated by GS algorithm and PPLL on 10 nm Au film

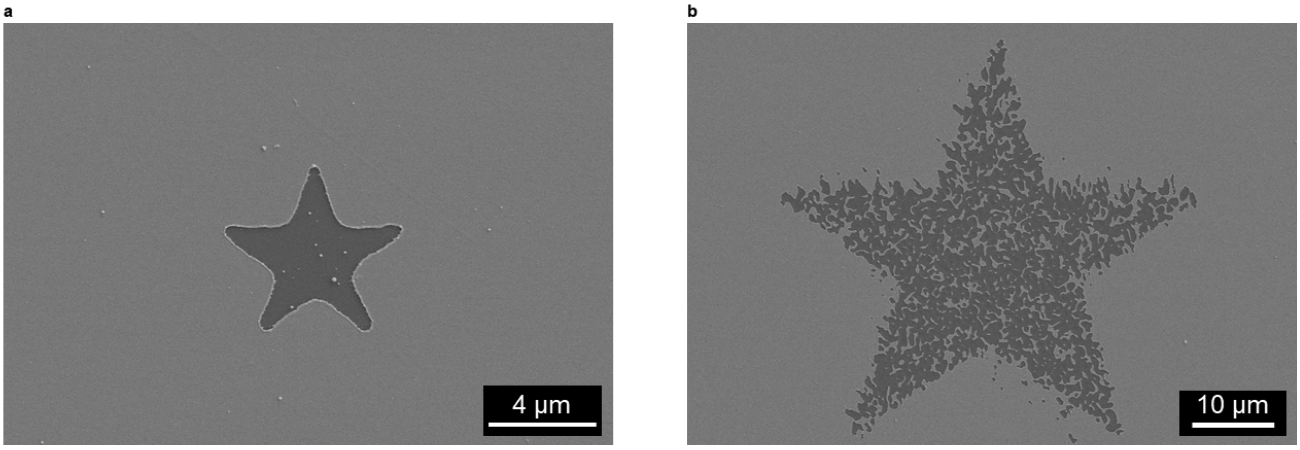

Fig. 1 The comparison of star structures fabricated by PPLL and CGH with the same objective. **a,** SEM image of the star structure fabricated by PPLL. **b**, SEM image of the star structure fabricated by CGH calculated by GS algorithm showing apparent speckle noise in laser energy distribution.

2. Principle of the PPLL

As shown in Fig. S2, the modulation undergoes several steps of change on phase, polarization, and intensity. A general 2D rectangular coordinate system is applied, whose normal direction is the same as the direction of the incident light. At first, the horizontally polarized light goes through a half-wave plate (with the fast axis rotation of –22.5°) to obtain a linear polarization of –45°, as expressed by Jones' matrix:

$$J_{②} = \begin{bmatrix} 1 \\ -1 \end{bmatrix} \qquad \text{(Eq. S1)}$$

Then the beam obtains phase delay from SLM by reflection resulting in a phase-delay-related elliptical polarization distribution:

$$J_{\mathrm{SLM}} = \begin{bmatrix} -e^{-i\sigma} & 0 \\ 0 & 1 \end{bmatrix} \qquad \sigma = 2(n_e - n_o)k\Delta z \qquad \text{(Eq. S2)}$$

$$J_{③} = J_{\mathrm{SLM}} \times J_{②} \Rightarrow \begin{bmatrix} e^{-i\sigma} \\ 1 \end{bmatrix} \qquad \text{(Eq. S3)}$$

where σ is the phase delay caused by the rotating biaxial liquid crystal pixel, the 8-bit grayscales of images projected on the SLM correspond to the phase delay from 0 to π. The generated polarization distribution is composed of a continuous change of elliptical polarization (Fig. S2c). It corresponds to the gradation of grayscales, which could help smooth the image boundary, flatten the intensity, and reduce the minimum ablation width.

After that, we use a quarter waveplate (with a fast axis of 45° tilted) to change the elliptical polarization into a rotating linear polarization (Fig. S2d) and introduces the image information into a rotating angle that is expressed as:

$$J_{④} = J_{\frac{1}{4}\,\mathrm{waveplate}} \times J_{③} = \begin{bmatrix} e^{-i\sigma} - i \\ -ie^{-i\sigma} + 1 \end{bmatrix} \Rightarrow e^{-i\left(\frac{\sigma}{2}+\frac{\pi}{4}\right)} \begin{bmatrix} \cos\left(\frac{\pi}{4} - \frac{\sigma}{2}\right) \\ \sin\left(\frac{\pi}{4} - \frac{\sigma}{2}\right) \end{bmatrix} \qquad \text{(Eq. S4)}$$

where the resulted Jones matrix denotes a polarization distribution with angles of $\pi/4$-$\sigma/2$.

Finally, the light is filtered by the polarization filter (with a polarization direction of 45° tilted) to

remain in one direction of the polarization. So that the light with different polarization directions would retain different strength with the same polarization, namely conducting the image information into light intensity (Figs. S2b,e). With the same polarization, the light intensity is given by:

$$J_{⑤} = J_{\text{polarization}} \times J_{④} = \begin{bmatrix} 1 & 1 \\ 1 & 1 \end{bmatrix} e^{-i\left(\frac{\sigma}{2}+\frac{\pi}{4}\right)} \begin{bmatrix} \cos\left(\frac{\pi}{4}-\frac{\sigma}{2}\right) \\ \sin\left(\frac{\pi}{4}-\frac{\sigma}{2}\right) \end{bmatrix} \Rightarrow \cos\frac{\sigma}{2} e^{-i\left(\frac{\sigma}{2}+\frac{\pi}{4}\right)} \begin{bmatrix} 1 \\ 1 \end{bmatrix} \qquad (\text{Eq. S5})$$

where the resulting matrix represents a linearly related intensity to cos(σ/2). Moreover, the QWP2 converts the linear polarization to the circular polarization for better ablation results with reasons explained in the section of "Uniform ablation by considering polarization-dependent effects and isotropy of structures" in the body text.

So far, we have obtained a direct relationship between phase delay from the patterned grayscale phase mask and the light intensity, which helps to conduct the pattern information from image view to light intensity. The results in Fig. S2e could be further modulated by waveplate to reach patterned intensity with various uniform polarization.

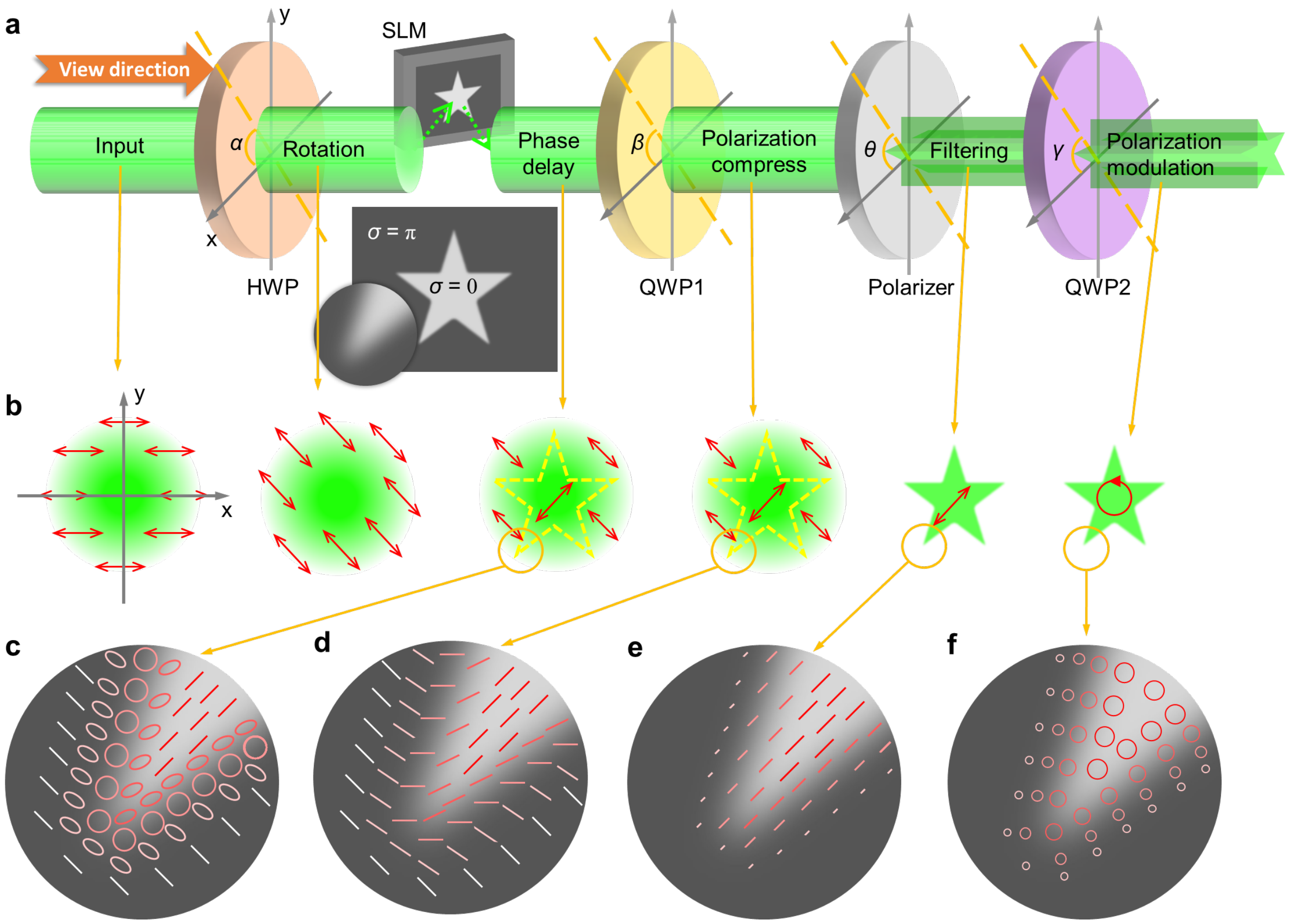

Fig. 2 Detailed light modulation mechanisms show the tailoring of light intensity distribution through filtering of polarizations. a, Optical system of the beam modulation process. The angle α, β, θ and γ refer to the angles between the fast axis and the positive x-axis direction, where $\alpha = -22.5°$, $\beta = 45°$, $\theta = 45°$, $\gamma = 90°$. **b,** The polarization and intensity distribution of the modulated beam at each position along the light path. The red arrows indicate the polarization direction of laser spots. **c-f**, Polarization evolution at the gradient-grayscale-level boundary.

3. The “L” shaped structure reveals a reduction of linewidth with binary phase masks with gradient boundary

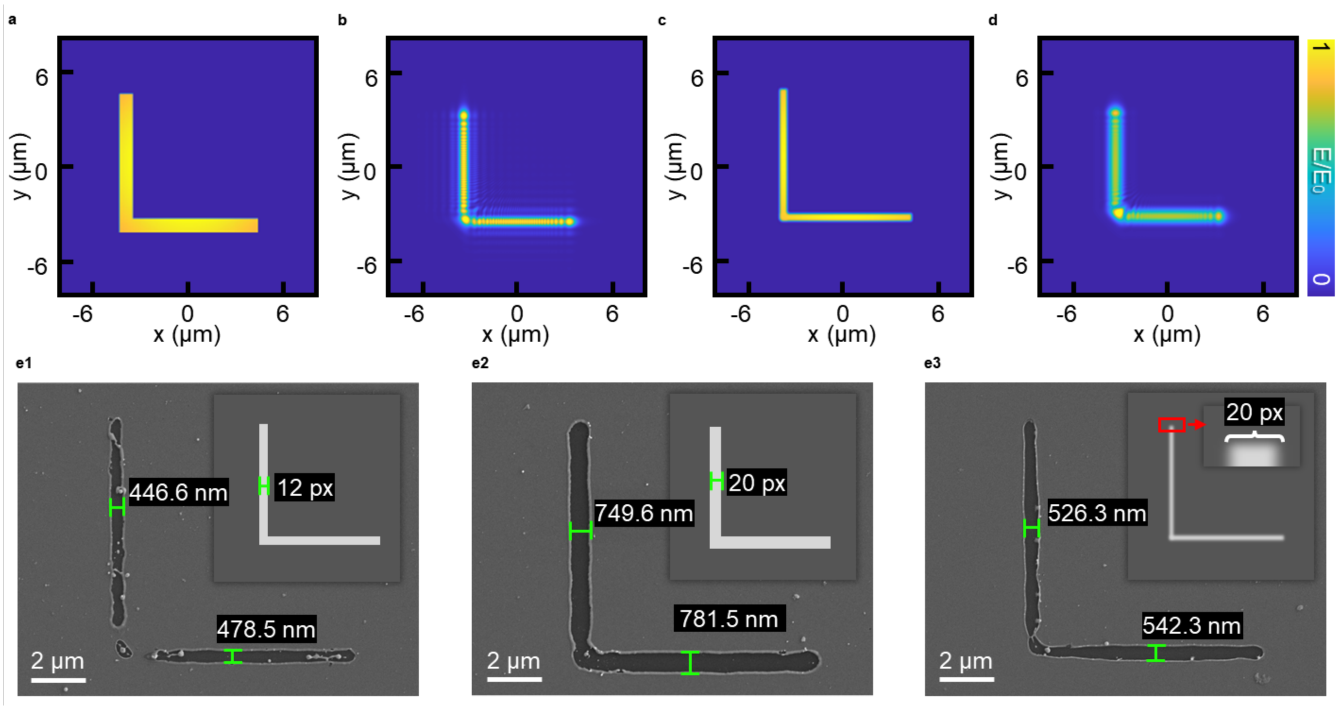

Fig. 3 a,**b**,**c**,**d**, Propagation calculation of “L” shape structure revealing smoother and finer center region for ablation with binary phase masks with gradient boundary. The ablation results are produced under objectives of NA = 0.55 and laser pulse energy is 0.14 µJ, 0.15 µJ and 0.14 µJ (with the same pulse energy before the SLM). **e1,** with 12-pixel linewidth, **e2**, with 20-pixel linewidth, and **e3**, with 20-pixel linewidth and 6-pixel grayscale gradation in each boundary.

A sharp “L” shape with a width of 12 pixels cannot be ablated completely. Under the same laser pulse energy, a sharp “L” shape with a width of 20 pixels could be ablated totally. By comparison, the boundary-gradient counterpart of 12-pixel lines (6-pixel gradation on each boundary) is feasible in ablation and even achieves smaller line width than a 20-pixel sharp one. Referring to the calculation results, the gradient boundary helps accumulate the light intensity to the center of the line where stronger ablation happens. So that, grayscale-gradient mask boundary can further improve the linewidth resolution beyond the diffraction limit. It is noticed that we have to choose the proper width

of gradation in consideration of feature sizes and pattern shapes. Here we select a relatively small gradation width (6 pixels) to preserve the sharp shape as much as possible.

4. The first Rayleigh-Sommerfeld diffraction integral under vector situation.

$$U_x(x_2, y_2, z) = \frac{i\exp(-ikz)}{\lambda z}\iint_{-\infty}^{+\infty} U_x(x_1, y_1, z)\exp\left\{-\frac{ik}{2z}[(x_2 - x_1)^2 + (y_2 - y_1)^2]\right\} dx_1 dy_1 \text{(Eq. S6)}$$

$$U_y(x_2, y_2, z) = \frac{i\exp(-ikz)}{\lambda z}\iint_{-\infty}^{+\infty} U_y(x_1, y_1, z)\exp\left\{-\frac{ik}{2z}[(x_2 - x_1)^2 + (y_2 - y_1)^2]\right\} dx_1 dy_1 \text{(Eq. S7)}$$

$$U_z(x_2, y_2, z) = \frac{i\exp(-ikz)}{\lambda z}\iint_{-\infty}^{+\infty} \left[U_x(x_1, y_1, z) \times (x_2 - x_1) + U_y(x_1, y_1, z) \times (y_2 - y_1)\right]$$

$$\exp\left\{-\frac{ik}{2z}[(x_2 - x_1)^2 + (y_2 - y_1)^2]\right\} dx_1 dy_1 \qquad \text{(Eq. S8)}$$

5. Calculation of the diffraction limit and the principle of zooming-out patterned laser spots

PPLL can produce patterns with sub-diffraction-limit feature size due to zooming out of the patterns by a 4f-system and the certain ablation thresholds that only the center of the Gaussian beam could ablate samples. The diffraction-limit width could be given by:

$$d_{\mathrm{limit}} = \frac{0.61\lambda}{NA} = \frac{0.61 \times 520\ \mathrm{nm}}{0.55} \approx 577\ \mathrm{nm} \quad (\mathrm{Eq.\,S9})$$

$$d_{\mathrm{limit}} = \frac{0.61\lambda}{NA} = \frac{0.61 \times 520\ \mathrm{nm}}{0.8} \approx 397\ \mathrm{nm} \quad (\mathrm{Eq.\,S10})$$

The experimental results show that the linewidths are smaller than diffraction-limit widths of objectives with NA of 0.55 (50× objective) and 0.8 (100× objective), especially the much smaller linewidths of ring shapes. Thus, the PPLL is a feasible approach to achieve structures with resolution smaller than the diffraction limit.

The ratio of zooming out of the patterned spot is proportional to the ratio of f_1 to f_2 (Fig. S4a), which is tunable by using different objectives and lenses with different focal lengths (Figs. S4c,d). When the laser is imposed on samples, only the region with laser fluence above the ablation threshold could be ablated.

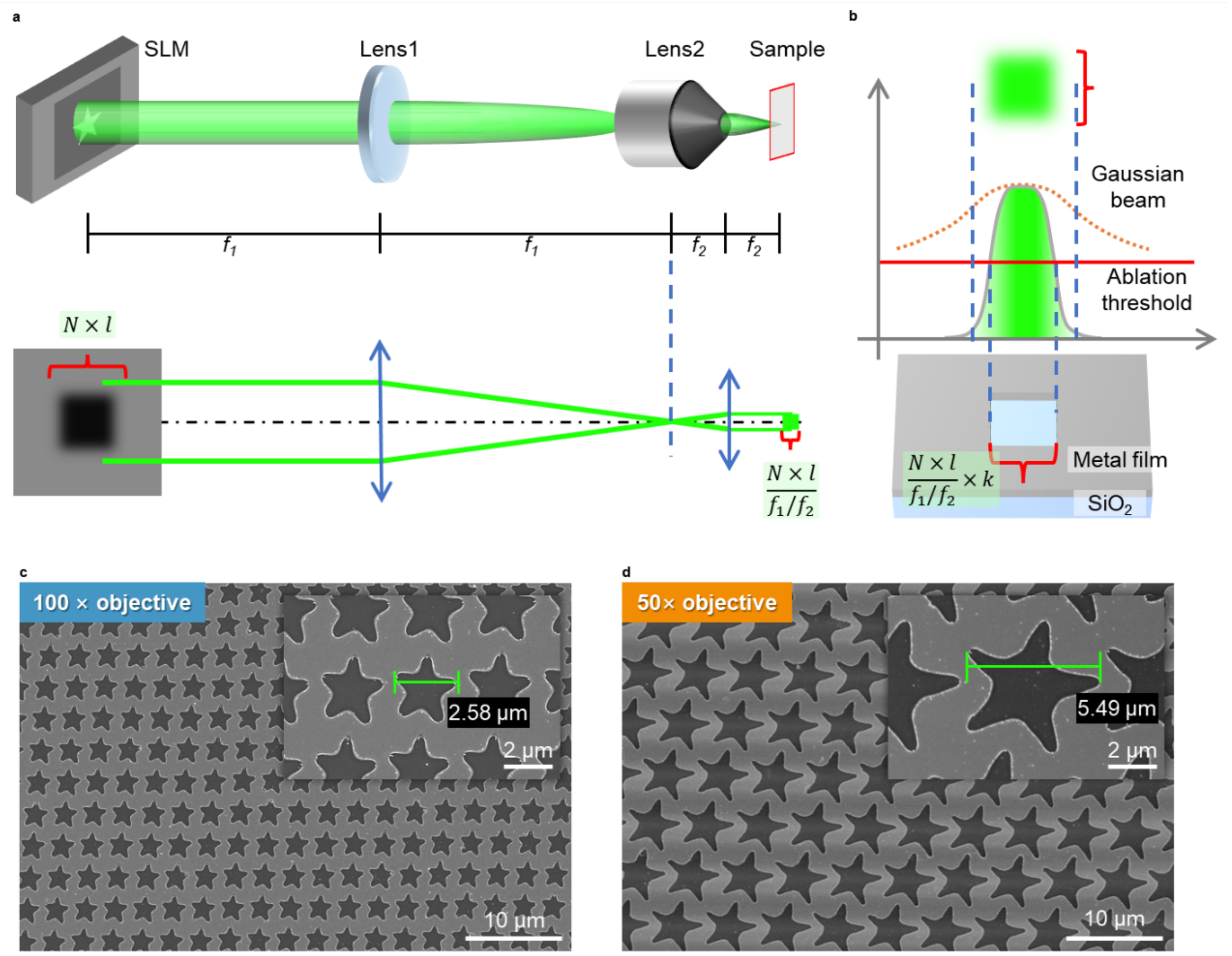

Fig. 4 Schematic of the 4f-system-based zooming-out principle of patterned spots. a, Schematic of the 4f system (f_1 = 1000 mm, f_2 = 4 mm for 50× objective or f_2 = 2 mm for 100× objective). The other waveplates are hidden to emphasize the 4f system. **b**, Schematic of the ablation process. **c**,**d**, Corresponding structure arrays fabricated by 100× and 50× objectives using the same phase mask. The results are produced on the samples composed of 10 nm Au, 3 nm Cr on surface of SiO_2 substrate.

6. Other details of structures fabricated by PPLL.

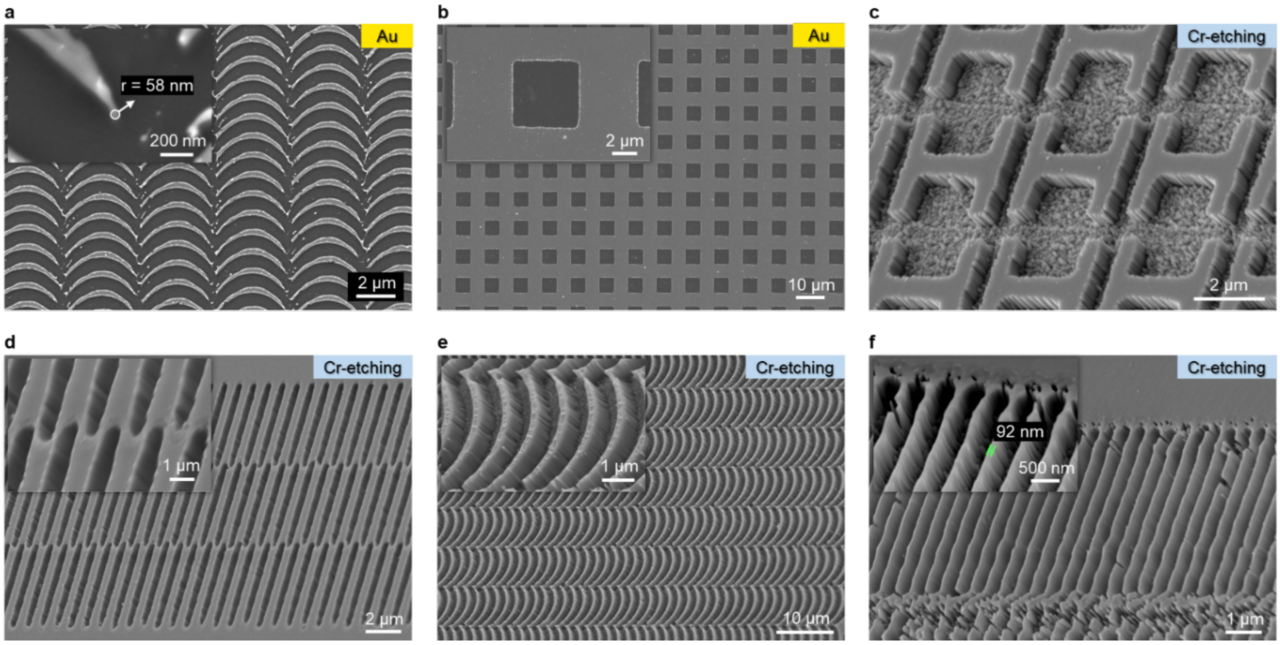

Fig. 5 Various structures fabricated by PPLL with more details. a,**b**, Demonstration of PPLL to fabricate catenaries and 2D gratings. **c**, "H" structures. **d**, Etching results of gratings with half-period mismatch compared with Fig. 6g. **e**,**f**, Catenaries and gratings with finer linewidth. "Au" for samples comprising 10 nm Au, 3 nm Cr on SiO_2 substrate. "Cr-etching" for samples comprising 30 nm Cr on SiO_2 substrate.

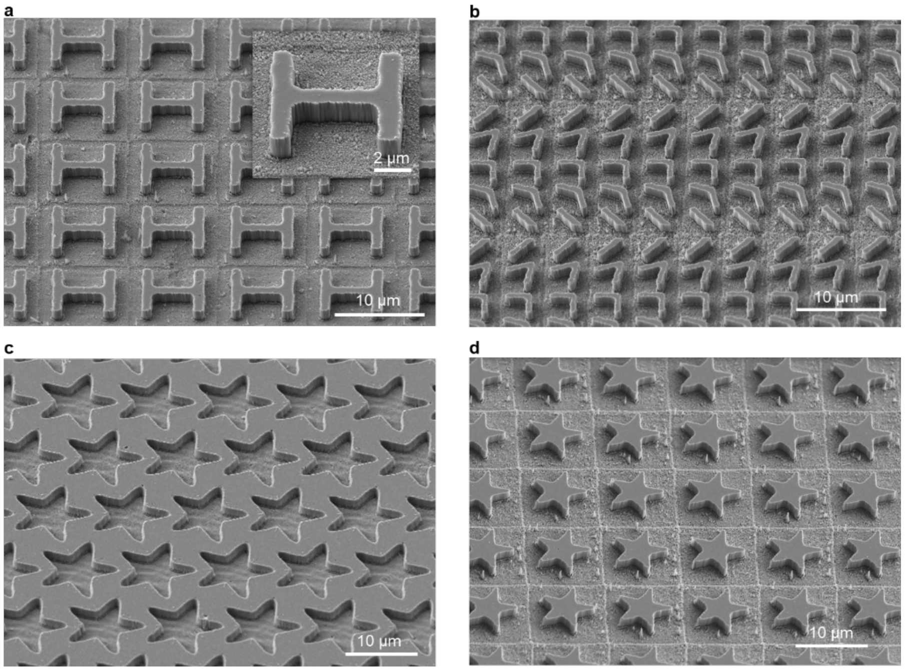

Fig. 6 Various results fabricated by PPLL with further etching process (50× objective). a, "H" shapes. **b**, Antennas. **c**, Star patterns. **d**, Reverse star patterns. The masks are patterned Cr with thickness of 30 nm on SiO_2 substrate.

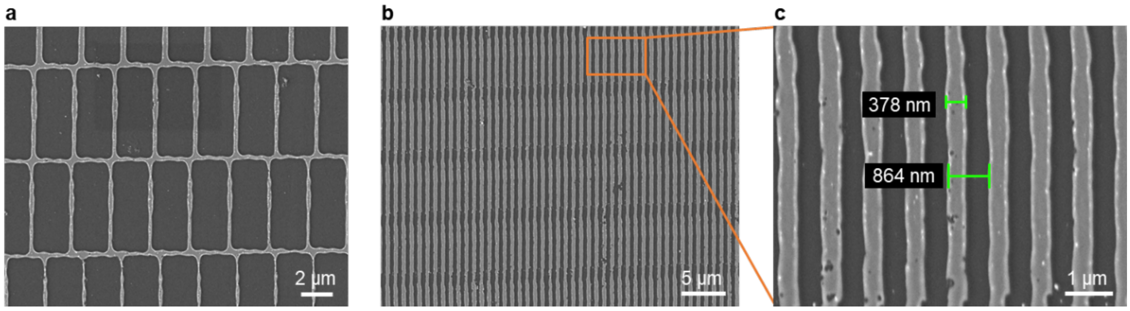

Fig. 7 Brick-like frames and large-are gratings produced by line pulse ablation. a, Brick-like grating mesh with pulse energy of 0.3 μJ. **b**,**c**, Long-range ordered gratings with enlarged image by pulse energy of 0.09 μJ.

7. Structural arrays with various distributions.

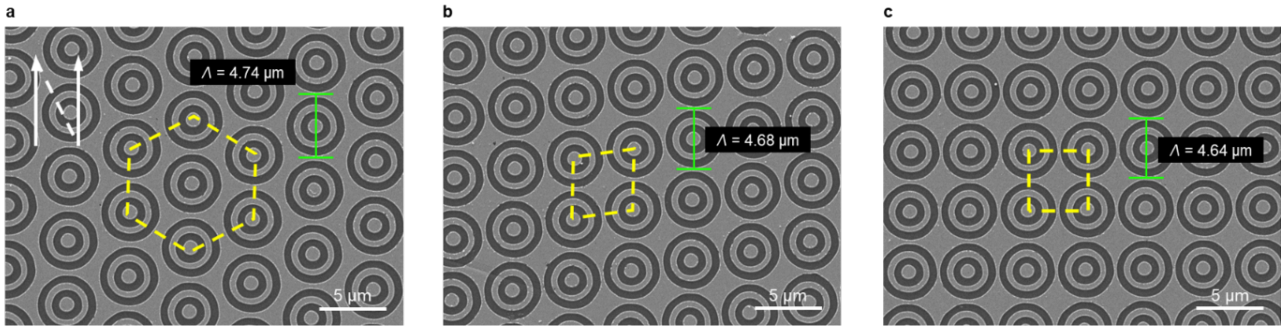

Fig. S8 Fabrication of structure arrays by tuning scanning strategy under 10 kHz. a, Vertical line scanning with a speed of 47.5 mm/s. **b**, Vertical line scanning with a speed of 47 mm/s. **c**, Vertical line scanning with a speed of 46.5 mm/s. All are produced by pulse energy of 0.23 μJ. Slight scanning speed change results in small period change but a massive difference in distribution of structural units under stable laser shutter device, revealing further controllability of PPLL.

8. The cross-sectional profiles of concentric structures on GST after etching.

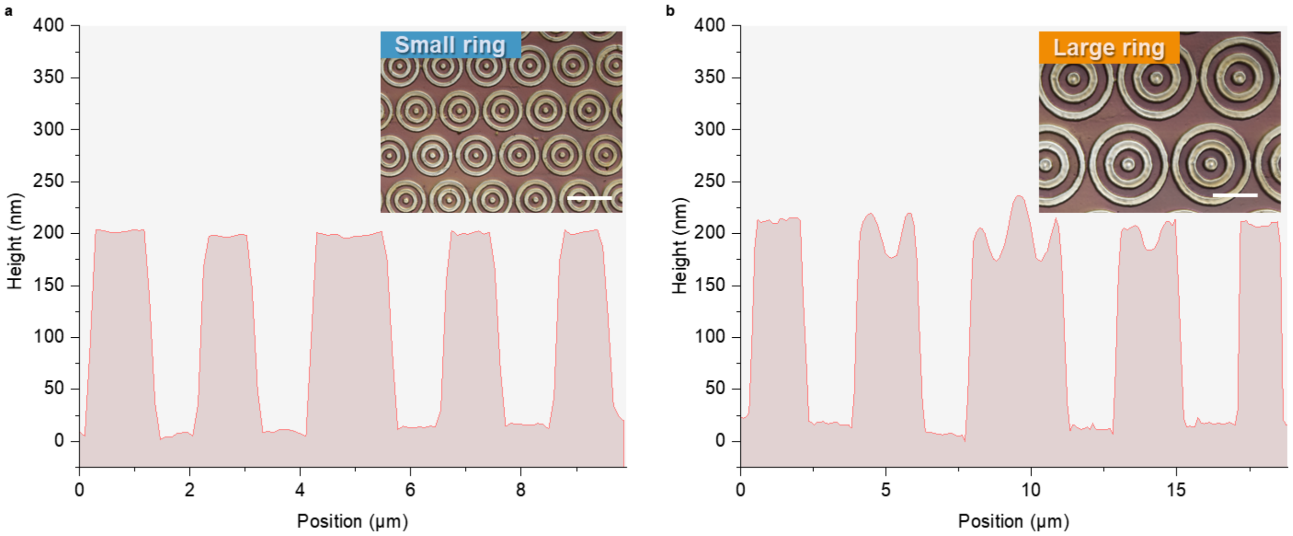

Fig. 9 The AFM results showing cross-sectional profiles of patterned structures on GST film after etching. a, Small rings. **b**, Large rings. Inset, the surface morphology of two types of concentric rings. Scale bars on the inserted images are 10 μm.

Table. 1 The depths of each layer of the absorber.

**Components**	GST_c	GST_a	Au	Cr	SiO_2
**Thickness (μm)**	0.19	0.05	0.07	0.005	0.5
